# Fractures sustained by slipping on ice or snow: an epidemiological study of 50,500 fractures from the Swedish Fracture Register

**DOI:** 10.2340/17453674.2025.43186

**Published:** 2025-03-21

**Authors:** Henrik IVDAL, Linnea BERGENHOLTZ, Carl BERGDAHL, Olof WOLF, Emilia Möller RYDBERG

**Affiliations:** 1 Institute of Clinical Sciences, Sahlgrenska Academy, University of Gothenburg, Gothenburg; 2 Department of Orthopaedics, Sahlgrenska University Hospital, Gothenburg/Mölndal; 3 Section of Orthopaedics, Department of Surgical Sciences, Uppsala University, Uppsala; 4 Department of Orthopaedics, Uppsala University Hospital, Uppsala, Sweden

## Abstract

**Background and purpose:**

Despite numerous patients sustaining fractures annually due to slipping on ice or snow, descriptive studies are scarce, which may result in less systematic management and prevention. We aimed to analyze fractures in adults sustained by slipping on ice and snow in Sweden using data from the Swedish Fracture Register (SFR).

**Methods:**

Data on all patients ≥ 18 years registered in the SFR between January 1, 2015 and December 31, 2022, with a fracture sustained by slipping on ice or snow was extracted. Descriptive statistical analyses were performed.

**Results:**

During the study period, 50,500 fractures were registered as sustained by slipping on ice or snow, representing 9% of all registered fractures in the SFR during the same period. 60% of the fractures affected the upper extremity. The mean age at the time of fracture was 61 years (18–105) and almost 70% of fractures were seen in women. The most common fractures were to the wrist (34%), ankle (18%), proximal humerus (11%), and hip (10%).

**Conclusion:**

Almost 1 in 10 fractures is sustained by slipping on ice or snow. The most common fractures are related to the wrist and the ankle. The majority of fractures affect the upper extremity, and two-thirds are sustained by women. Protective shoe wear, and better snow and ice clearance, could potentially have a large effect on injury prevention.

Like many other countries, Sweden has large seasonal weather variations and 4 distinct seasons. During the winter (November to March) the entire country can be affected by snow and ice but there is great variation in duration and character. In the more densely populated areas in the middle and south, slippery conditions are generally due to ice because temperatures are shifting around freezing point, whereas snow often covers the ground throughout the winter in the north.

Amongst orthopedic surgeons it is common knowledge that ice and snow cause an increased inflow of patients with fractures [1-6]. However, the fracture distribution sustained by slipping on ice and snow is not well studied and the few scientific studies examining this are based on small cohorts. Previous publications from the Swedish Fracture Register (SFR) on ankle, wrist, and proximal humerus fractures have demonstrated a pronounced seasonal variation with an increase in fractures during the winter months [7-9]. A similar seasonal variation in hip and forearm fractures has been shown from Great Britain and Norway but the full picture of fracture distribution in conditions with ice and snow remains unclear [10-11].

We aimed to analyze fractures sustained by slipping on ice and snow from a nationwide perspective in Sweden with regards to distribution of fracture types, sex, and age.

## Methods

### Study design

This is an observational register study on data from the SFR. Data was extracted on all patients ≥ 18 years at injury and registered in SFR with a fracture sustained by slipping on ice or snow between January 1, 2015 and December 31, 2022.

### Population

Both patients with 1 isolated fracture and patients with multiple fractures were analyzed. Analyses were made for age at the time of injury, sex, fracture localization, open or closed fracture, and high- or low-energy injury. Fracture locations were grouped into wrist (fractures to the distal radius and distal ulna), ankle (fractures involving the malleoli), proximal humerus (fractures to the proximal humerus), hip (cervical, trochanteric, and subtrochanteric fractures), elbow (proximal radius and ulna, distal humerus), hand (all hand fractures), knee (distal femur, proximal tibia, and patella), pelvis (acetabulum and pelvis), shoulder (scapula and clavicle), spine (all spine fractures), and other (foot, diaphyseal fractures to the femur, humerus, tibia and lower arm, and fracture to the distal tibia).

The manuscript was written following the STROBE guidelines for observational studies.

### Swedish Fracture Register

The SFR is a web-based national quality register that holds information on fractures, injury mechanism, and treatment. Following its inception in 2011 more than 1,000,000 fractures had been registered by October 2024. All Swedish hospitals providing trauma care are enrolled in registering fractures since 2021, resulting in full coverage. Completeness has been found to be 58% for all fracture types when compared with the National Patient Register (NPR), but the true completeness is probably higher as the NPR overrates the number of fractures because of follow-up visits [12]. Registrations are made by the physician and contain information regarding injury mechanism and fracture classification. Moreover both surgical and non-surgical treatment are reported [13,14]. The classification system used is mainly the 2007 Arbeitsgemeinschaft für Osteosynthesefragen (AO) foundation/Orthopedic Trauma Association (AO/OTA) classification [15]. Registration of injury mechanism is done by the physician in a step-wise menu tree starting with injury date, injury type, injury location, and high- or low-energy trauma mechanism, prior to the AO/OTA fracture classification, explained in detail in prior publications [13,14]. Several studies have proven good validity of the classification data in the SFR [16-18].

### Statistics

Descriptive statistics for categorical data were presented as count and proportion (%) and for numerical data as mean (range). Subgroup analyses included sex and age groups (18–30, 31–40, 41–50, 51–60, 61–70, 71–80, 81–90, 91–100, 101 years and older). Age, sex, fracture localization, number of fractures, open or closed fracture, and high- or low-energy injury are presented using descriptive statistics. All statistics in the study were calculated using the software IBM SPSS statistics v 29 (IBM Corp, Armonk, NY, USA).

### Ethics, registration, data sharing plan, funding, and disclosures

The study was approved by the Swedish Ethical Review Authority (2022-04355-01). All patients were informed at registration in the Swedish Fracture Register that they had the right to withdraw. According to Swedish legislation, National Quality Registers do not require signed consent from the individual registered patient. The datasets used and analyzed during the current study are available from the corresponding author on reasonable request. The research methods were carried out in accordance with the Declaration of Helsinki. No funding was received. For all authors, no conflicts of interest were declared. Complete disclosure of interest forms according to ICMJE are available on the article page, doi: 110.2340/17453674.2025.43186

## Results

**Figure 1:**
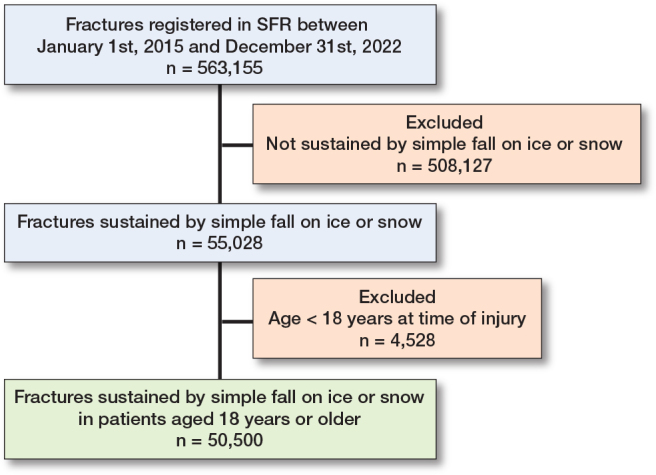
Flowchart of patient inclusions.

563,155 fractures in adults were registered with the SFR in study period (Figure 1). 50,500 fractures in 48,440 patients were sustained by falls on ice and snow, representing 9% of all fractures. 1,929 (4%) patients sustained 2 or more fractures, either on the same injury occasion or on different injury occasions during the study period. Women were more often affected than men (69% and 31% of the fractures respectively). The mean age at the time of injury was 61 years (18–105), with a higher mean age for women (63 years) than men (58 years). 1% of the fractures were open and 1% were registered as high-energy injuries (Table 1).

**Table 1:** Demographics for fractures sustained after falling or ice or snow, stratified by type of fracture. Values are count (%) unless otherwise specified

Factor	All fractures	Wrist	Ankle	Proximal humerus	Hip	Elbow	Hand	Knee	Pelvis	Shoulder	Spine	Other
Fractures	50,500	17,159 (34)	9,180 (18)	5,395 (11)	5,086 (10)	3,085 (6.1)	2,948 (5.8)	1,634 (3.2)	1,189 (2.4)	1,145 (2.3)	720 (1.4)	2,763 (5.5)
Sex												
Male	15,819 (31)	2,699 (16)	3,642 (40)	1,268 (24)	2,311 (45)	884 (29)	1,619 (55)	543 (33)	440 (37)	728 (64)	374 (52)	1,233 (45)
Female	34,681 (69)	14,460 (84)	5,538 (60)	4,127 (77)	2,775 (55)	2,201 (71)	1,329 (45)	1,091 (67)	749 (63)	417 (36)	346 (48)	1,530 (55)
Age, mean (range)												
Total	61 (18–105)	62 (18–100)	56 (18–98)	67 (18–104)	76 (21–105)	54 (18–97)	51 (18–97)	61 (18–98)	70 (19–100)	55 (18–103)	66 (19–99)	55 (18–99)
Male	58 (18–105)	59 (18–99)	54 (18–98)	64 (18–96)	75 (21–105)	50 (18–97)	46 (18–97)	57 (18–94)	70 (20–96)	51 (18–97)	66 (19–97)	52 (18–97)
Female	63 (18–104)	62 (18–100)	57 (18–96)	68 (18–104)	77 (22–104)	56 (18–95)	57 (18–95)	63 (18–98)	70 (19–100)	63 (18–103)	66 (19–99)	57 (18–99)
Fracture type												
Open	389 (0.8)	146 (0.9)	83 (0.9)	1 (0)	2 (0)	35 (1.1)	15 (0.5)	8 (0.5)	0 (0)	2 (0.2)	0 (0)	97 (3.5)
High energy	660 (1.3)	149 (0.9)	105 (1.1)	66 (1.2)	38 (0.7)	41 (1.3)	40 (1.4)	63 (3.9)	13 (1.1)	39 (3.4)	24 (3.3)	69 (2.5)
Low energy	43,637 (86)	14,981 (87)	7,902 (86)	4,641 (86)	4,525 (89)	2,660 (86)	2,536 (86)	1,345 (82)	1,036 (87)	950 (83)	582 (81)	2,336 (85)

Missing values for type of fracture: 196 (0.4%). For high- and low-energy injuries, data is not shown for cases where this variable was registered as “unknown” or “not applicable.”

### Fracture, age, and sex distribution

**Figure 2:**
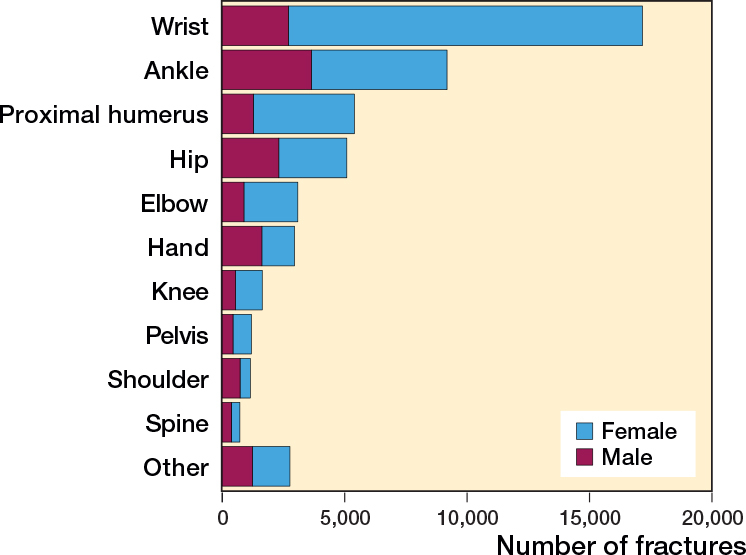
Distribution of fractures sustained by falls on ice of snow by fracture type and sex.

The most common fracture sustained were wrist fractures (34%), followed by fractures to the ankle (18%), proximal humerus (11%), and hip (10%) (Figure 2).

Women comprised the vast majority in the common fracture types like wrist fractures (84% women), proximal humerus (77%), and elbow (71%). A slight male dominance was seen in the more uncommon fracture types like fractures to the shoulder (64% men), hand (55%), and spine (52%) (Table 1, Figure 2).

The majority of fractures affected the upper extremity (60%). Over 75% of the upper extremity fractures were sustained by women (Table 2).

**Table 2:** Demographics of fractures sustained by falling on ice or snow stratified by body part

Body part	Fractures, n (%)	Mean age (range)	% female
Upper extremity	30,413 (60)	60 (18–104)	75
Lower extremity	19,171 (38)	62 (18–105)	59
Spine	720 (1.4)	66 (19–99)	48

**Figure 3:**
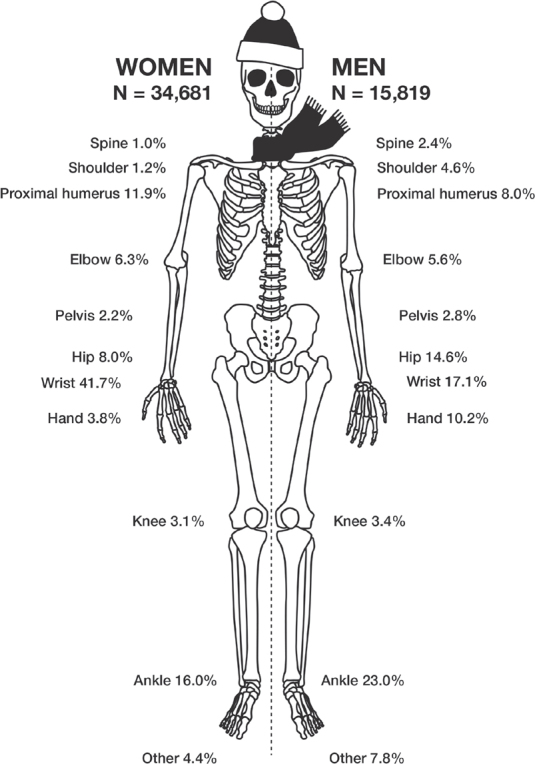
Distribution between most common fracture types sustained after falling on ice or snow, stratified by sex.

There were pronounced differences between men and women in the fracture distribution. For women the most common fracture was a wrist fracture, representing 42% of all fractures. The second most common fracture in women was a fracture to the ankle (16%) followed by a fracture to the proximal humerus (12%). For men the most common fracture type was a fracture to the ankle (23%), followed by the wrist (17%), and the hip (15%) (Figure 3).

The highest mean age at the time of injury was seen for hip fractures (76 years) and the lowest mean age in hand fractures (51 years) (Table 1).

**Figure 4:**
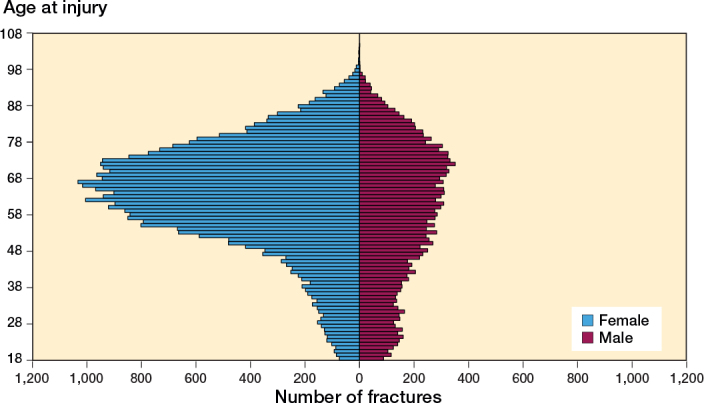
Age and sex distribution of fractures sustained in falls on ice or snow.

Both sexes demonstrated a unimodal age distribution with a peak of fractures seen between 50 and 80 years. The peak was more pronounced for women while men had a flatter curve at both ends. Moreover, an additional small peak was seen in men between the ages of 18 to 30 years, which was not seen for women (Figure 4).

### The 4 most common fracture types

**Figure 5:**
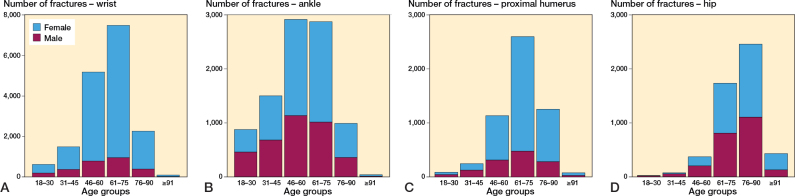
Distribution of fractures among the age groups for the 4 most common types of fractures sustained after falling on ice or snow: (A) wrist, (B) ankle, (C) proximal humerus, (D) hip.

*Wrist fractures.* 84% of the wrist fractures affected women and women were in the majority in all age groups. The distribution between age groups demonstrated a distinct peak in the age group of 61–75 years, with a pronounced increase of fractures starting at the age of 46 and followed by a clear decline after the age of 76 years (Figure 5A).

*Ankle fractures.* There was a different age and sex distribution in ankle fractures compared with the other 4 most common fracture types. A more even distribution between the sexes was seen throughout the age groups. Moreover, a peak was seen for the age groups 46–75 years but the curve is flatter and involves all age groups from 18 to 90 years (Figure 5B).

*Proximal humerus fractures.* A very similar distribution between the age groups and sexes was seen for the wrist and the proximal humerus fractures. However, compared with the wrist fractures, fractures of the proximal humerus demonstrated a distinct peak between the age of 61 and 75 years (Figure 5C).

*Hip fractures.* The hip fracture patients had a mean age of 76 years at the time of injury and 55% of the fractures were in women. The peak for hip fractures occurred later than for the other common fracture types, with a clear increase from 61 years and a peak in the age group 76–90 years (Figure 5D).

## Discussion

We aimed to analyze fractures sustained by slipping on ice and snow in adults in Sweden. We found they represented almost 1 in 10 fractures nationally in Sweden and primarily affect women. The most common fractures were to the wrist and the ankle. Wrist fractures constituted over one-third of all fractures sustained after slipping on ice or snow. The fracture panorama after a fall on ice and snow differs from the general fracture panorama in the larger proportion of wrist and ankle fractures and the increased proportion of men sustaining hip fractures.

Slipping on ice and snow is a well-known cause of orthopedic injury and an increase in inflow of orthopedic patients on days with ice and snow has been observed [4,6,19-21]. There are several previous studies mapping the incidence of specific fractures [7,8,22] but few studies explore the panorama of fractures caused by a specific injury mechanism [23-25]. Only a few previous studies have examined injuries related to ice and snow events. A Canadian study reporting on 3,894 patients from a single center demonstrates a similar sex and age distribution to our study with 70% women and a mean age of 51 years. In a study from 1998, Smith et al. reported that 62% of the patients sustaining fractures following an ice-storm were women and that the mean age was 42 years [20]. The difference in mean age compared with the findings in our study (mean age 61 years) is probably explained by the inclusion of children in these studies. As mentioned, the previous studies are on small volumes of material and from a single center. Our study is to our knowledge by far the largest mapping of fractures sustained by falls on ice or snow and also has national coverage and a study time of several years.

The findings regarding the distribution of fractures in our study differ in the proportion of the most commonly sustained fractures compared with previous studies on the epidemiology of fractures sustained by all injury mechanisms. A study by Bergh et al. reporting on fracture incidence for all injury mechanisms in a larger region in Sweden concluded that the 5 most common types of fractures were fractures to the distal radius (16%), proximal femur (15%), ankle (10%), proximal humerus (8%), and metacarpal bones (7%) (26). In our study 4 of these 5 fractures are also found to be most common but the proportion of primarily wrist and ankle fractures was found to be much higher.

Trauma care is difficult to plan and even more so when resources are scarce. It has been shown that the weather does influence the workload in emergency departments and the need for orthopedic surgical resources. Every year the authorities and media in Sweden issue warnings regarding perilous conditions and the risk of slipping on ice and snow is deemed to be high. This is one of the preventive measures suggested in the literature [1,19,21]. Another measure is wearing proper shoes and anti-slip devices such as cleats or studded footwear [5,20,21,27]. However, poor quality, incorrect application, difficulties to apply, and awkward usage have been shown to lower the efficacy of such devices [27]. Society can provide snow clearing, sanding, and salting to reduce the risk of falls. We stress the need for preventive measures in the group of upper middle-age and elderly women foremost. A general awareness of when slippery conditions are present in the population might be of importance as well as other preventive measures mentioned and a relocation of resources in healthcare in times of ice and snow [21].

Patients with hip fractures due to slipping in icy conditions differ from the typical hip fracture patients described in previous studies [22,26,28]. In our study, 45% of the hip fractures affected men and the mean age was 76 years, compared with 31% men and a mean age of 82 years in the study by Mattisson et al. [22]. Hip fractures sustained in slippery weather conditions thereby affect a younger age group with a higher male prevalence than the general hip fracture panorama. Hip fractures have generally been regarded as a fracture not related to the weather conditions as they have been known to occur primarily indoors. We found that hip fractures were among the 4 most common fracture types sustained when falling on ice and snow even though the proportion was slightly lower than in the study by Bergh et al. on fractures caused by all injury mechanisms [26]. The finding that hip fractures are among the most common fracture types even when only fractures sustained by slipping on ice and snow are studied is supported by the findings of Smith and Nelson, where hip fractures were found to be the third most common fracture sustained in slippery conditions [20].

### Strengths

The biggest strength ofo this study is the unprecedented sample size of over 50,000 fractures sustained after a fall on ice or snow. Another strength is the study period of 8 consecutive years, making the impact of single especially snowy or icy winters less influential on the results. The study is also nationwide, covering all orthopedic departments registering fractures with the SFR thus enhancing the external validity of the study.

### Limitations

The injury mechanism has not been validated in the SFR. However, registering the injury mechanism “slipping on ice or snow” in the SFR is an active action in multiple steps, and our belief is that the risk of misclassifications in this regard is low. On the other hand, some fractures are probably missing from the study where the responsible physician did not actively register the fracture as caused by a fall “on ice or snow” but just as an unspecified same-level fall. The registration in the SFR regarding high-energy trauma is a less active action where this is selected from a drop-down menu, possibly explaining the 660 fractures registered as sustained by high-energy trauma. As slipping on ice and snow is registered as a same-level fall, which by definition cannot be high-energy trauma, these registrations are probably misclassifications but have been kept in the analyses for completion. Moreover, the proportion of these registrations is so small that it probably does not affect outcome. Another limitation is the completeness of registrations in the SFR, which is 58%. We do not believe that we have any systematic bias in reporting, as previous assessments of completeness in the annual reports from the SFR has demonstrated no regional differences regarding completeness,

### Conclusion

The most important finding of our study is that fractures sustained by falling on ice and snow primarily affect women and the most common fractures sustained are to the wrist and the ankle. Over 60% of the fractures sustained when falling on ice and snow are located in the upper extremity and the most common is a fracture to the wrist. Over 84% of the wrist fractures affect women and the peak starts as early as the age of 50 years at the time of injury. The patients sustaining a hip fracture from falling on ice or snow are younger and to a greater extent men than the general hip fracture patient.

*In perspective,* at a time where resources are scarce and waiting lists for orthopedic surgery and care are long, prevention of fall accidents in slippery conditions should be a priority, both from an economic and a fellow humanity perspective. Protective shoe wear, and better snow and ice clearance, could potentially have a substantial effect on injury prevention.

## Notes

HI planned and conducted the study and the statistical calculations, analyzed data, and wrote the first draft of the manuscript. EMR planned the study, conducted the statistical calculations, and revised the manuscript. OW wrote the ethical application and revised the manuscript. CB revised the manuscript.

The authors would like to thank all the orthopedic surgeons at the affiliated departments for entering detailed data in the Swedish Fracture Register on busy working days.

Handling co-editor: Bart A Swierstra.

*Acta* thanks Jan-Erik Gjertsen and Brynjólfur Jónsson for help with peer review of this manuscript.
